# Macrophage-derived ectosomal miR-350-3p promotes osteoarthritis progression through downregulating chondrocyte H3K36 methyltransferase NSD1

**DOI:** 10.1038/s41420-024-01986-5

**Published:** 2024-05-08

**Authors:** Rengui Lin, Jianbin Yin, Jialuo Huang, Liping Zou, Liangliang Liu, Wen Tang, Hongbo Zhang, Lingfeng Yang, Yu Zhang, Guangming Li, Guiqing Wang, Daozhang Cai, Haiyan Zhang, Yanli Liu, Yan Shao

**Affiliations:** 1 Department of Joint Surgery, Center for Orthopaedic Surgery, The Third Affiliated Hospital of Southern Medical University, Guangzhou, China; 2 Department of Orthopedics, Orthopedic Hospital of Guangdong Province, Academy of Orthopedics·Guangdong Province, The Third Affiliated Hospital of Southern Medical University, Guangzhou, China; 3 The Third School of Clinical Medicine, Southern Medical University, Guangzhou, China; 4 Guangdong Provincial Key Laboratory of Bone and Joint Degeneration Diseases, Guangzhou, China; 5 The Sixth Affiliated Hospital of Guangzhou Medical University, Qingyuan People’s Hospital, orthopedics department, Qingyuan, Guangdong, China

**Keywords:** Senescence, Osteoarthritis

## Abstract

Mechanical overloading can promote cartilage senescence and osteoarthritis (OA) development, but its impact on synovial macrophages and the interaction between macrophages and chondrocytes remain unknown. Here, we found that macrophages exhibited M1 polarization under mechanical overloading and secreted ectosomes that induced cartilage degradation and senescence. By performing miRNA sequencing on ectosomes, we identified highly expressed miR-350-3p as a key factor mediating the homeostatic imbalance of chondrocytes caused by M1-polarized macrophages, this result being confirmed by altering the miR-350-3p level in chondrocytes with mimics and inhibitor. In experimental OA mice, miR-350-3p was increased in synovium and cartilage, while intra-articular injection of antagomir-350-3p inhibited the increase of miR-350-3p and alleviated cartilage degeneration and senescence. Further studies showed that macrophage-derived ectosomal miR-350-3p promoted OA progression by inhibiting nuclear receptor binding SET domain protein 1(NSD1) in chondrocytes and regulating histone H3 lysine 36(H3K36) methylation. This study demonstrated that the targeting of macrophage-derived ectosomal miRNAs was a potential therapeutic method for mechanical overload-induced OA.

## Introduction

Osteoarthritis (OA) is a prevalent joint disease that significantly impacts the mobility and mental health of middle-aged and older people [1]. Its pathological changes are now widely recognized as low-grade inflammation involving the entire joint, including synovial inflammation and irreversible cartilage degeneration [2, 3**]**. Although the current epidemiology of OA shows that abnormal or excessive mechanical loading is closely related to cartilage degeneration, its etiology and pathogenesis are still to be elucidated [4, 5**]**.

Appropriate mechanical loading is necessary to maintain joint homeostasis under physiologic conditions [6]. Articular chondrocytes regulate extracellular matrix synthesis and maintain cartilage health by sensing and responding to mechanical signals in the joint microenvironment [7]. However, excessive mechanical loading induces mechanoinflammation in cartilage and activates inflammation-related signaling pathways, leading to catabolic enzyme production and the degradation of functional cartilage extracellular matrix [8, 9**]**. Therefore, correcting pathologic mechanical signal transduction or targeted inhibition of mechanoinflammatory signaling pathways in cartilage are potential treatments for OA. In joints, for synovial tissue also exposed to mechanical stimulation, studies have shown that high-frequency tensile strain induced synovial metabolic disorders and oxidative stress, resulting in reduced tolerance to mechanical loading in OA patients [10]. Macrophages are not only the most abundant immune cell type in OA synovial tissue, but are also mechanically reactive cells, which can alter their lineages according to the mechanical stimuli of the surrounding environment [11, 12]. Synovial macrophages can be induced to release of a variety of inflammatory factors when sensing changes in circulatory pressure, mediating osteolysis around joint replacement surgery, indicating that mechanical loading can mediate perisynovial tissue damage by regulating synovial macrophage activation [13, 14]. However, in OA progression, the functional transformation of synovial macrophages mediated by excessive mechanical loading and whether activated macrophages are involved in the regulation of cartilage degeneration is unclear.

Extracellular vesicles (EVs) are carriers for the horizontal transfer of substances and information between cells. They mediate proximal or distal cellular communication by transporting a variety of functional substrates associated with tissue homeostasis and disease [15, 16]. Recent research showed that macrophage-derived EVs led to cartilage catabolism and synovial inflammation, supporting the important role of EVs in OA [17]. Furthermore, synovial fibroblasts delivered microRNAs(miRNAs) to chondrocytes through EVs to mediate chondrocyte function, indicating that the EVs delivery function may be a potential mechanism in OA progression [18]. According to their different modes of biogenesis and release, EVs can be divided into exosomes (diameter 30–150 nm, formed through inward budding of the endosomal membrane), ectosomes (diameter 100–1000 nm, originated from the plasma membrane through outward budding), and apoptotic bodies (derived from terminal apoptotic cells) [19–21]. In recent years, with the expanding research on EVs subtypes, it has been found that the biochemistry and functions of ectosomes and exosomes were distinct [22–24]. Compared with exosomes, ectosomes from human hepatoma cells have greater capacity to promote monocyte differentiation, and their functions are associated with the glycolytic pathway [25]. Moreover, different from the metabolites in exosomes, metabolic molecules of ectosomes in pleural effusion can be used as candidate markers to distinguish tuberculosis from malignant tumors [23]. These findings revealed the important potential of ectosomes in cellular metabolism. However, previous research has mostly focused on exosomes in OA, whereas the molecular characteristics and functions of ectosomes remain unclear. Therefore, investigating the role of ectosomes may provide new approaches in OA diagnosis and treatment.

In our investigation, we observed that mechanical overloading caused the polarization of macrophages towards the M1 phenotype and its release of ectosomes that leads to chondrocyte catabolic disorders and senescence in vitro and in vivo. Through miRNA sequencing of ectosomes, we found that a high miR-350-3p abundance in ectosomes was a key factor leading to cartilage degeneration. In the mechanism, miR-350-3p regulated histone H3 lysine 36 (H3K36) methylation level by inhibiting nuclear receptor binding SET domain protein 1(NSD1) in chondrocytes, while H3K36 methylation plays an important role in cartilage development and differentiation [26]. Therefore, we hypothesized that the intercellular transfer of miR-350-3p mediated by synovial macrophage-derived ectosomes may be a potential OA pathogenesis mechanism under excessive mechanical loading.

## Results

### Ectosomes derived from mechanical overloading-stimulated macrophages induce cartilage catabolism and senescence

**Fig. 1:**
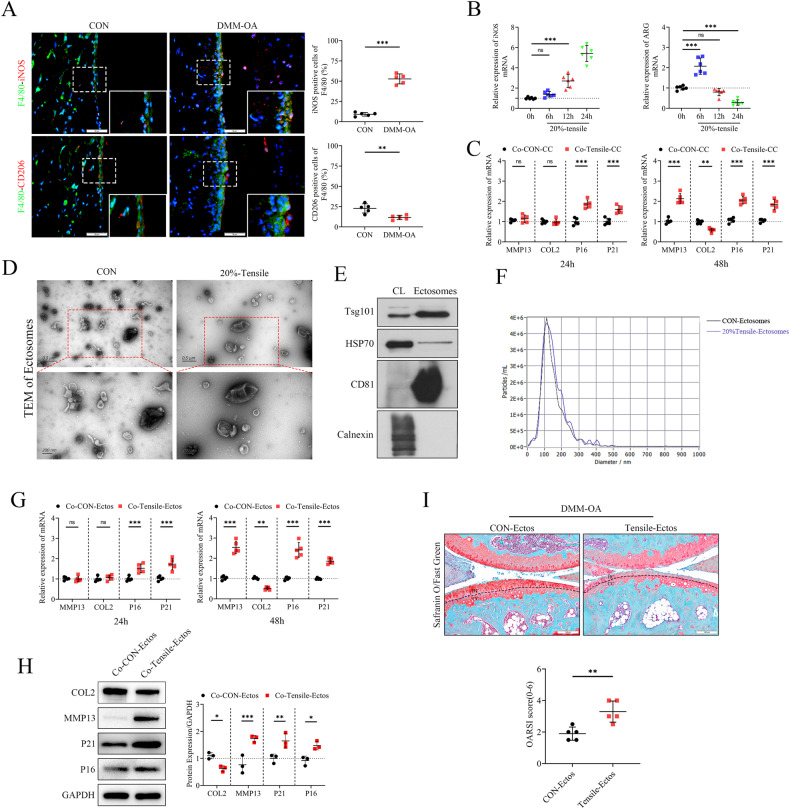
Ectosomes derived from mechanical overloading-stimulated macrophages induce cartilage catabolism and senescence. **A** Immunofluorescent (IF) staining of F4/80, CD206 and iNOS in synovium of sham-operated mice (CON) and DMM mice, *n* = 5 per group. Scale bar: 50 µm. **B** Quantitative PCR analysis of iNOS and ARG in Raw264.7 cells treated with 0.5 Hz, 20%-elongation strain loading for 0, 6, 12 and 24 h, *n* = 6 per point. **C** Quantitative PCR analysis of MMP13, COL2, P21 and P16 in chondrocytes co-cultured with supernatants of overloaded-RAW264.7 cells for 24 h or 48 h, *n* = 5 per group. **D** Morphology of ectosomes observed by transmission electron microscopy. Scale bar: 0.5 µm, 200 nm. **E** Western blot of ectosomes surface markers (performed by EchoBiotech Co. Ltd., Beijing, P. R. China). **F** Particle size distribution of ectosomes was measured by ZetaView nanoparticle tracking analysis. **G**, **H** Quantitative PCR and western blot of MMP13, COL2, P21 and P16 in chondrocytes treated with CON-Ectos or Tensile-Ectos for 24 h or 48 h. **I** Safranin O and Fast Green staining and OARSI grades of knee cartilage from DMM mice treated with CON-Ectos or Tensile-Ectos for 4 weeks, *n* = 5 per group. Scale bar: 100 µm. Statistical analyses were conducted by unpaired t-test (**A**, **I**), two-way analysis of variance followed by Sidak’s multiple comparison test (**C**, **G**) or one-way analysis of variance followed by Dunnett’s multiple comparison test (**B**). *P < 0.05, **P < 0.01, ***P < 0.001, ns not significant. OARSI Osteoarthritis Research Society International.

To explore the impact of mechanical overloading on the polarization of macrophages, we initially assessed the polarization status of synovial macrophages in a mechanical load-induced OA mouse model. Mouse synovial macrophages under mechanical loading induced by medial meniscal instability displayed accumulation of M1 polarization and decreased M2 polarization (Fig. 1A). Our previous study showed that 0.5 Hz, 20% cyclic tensile strain loading could lead to chondrocytes degeneration [27]. Therefore, we performed gradient cyclic tensile strain loading (0.5 Hz and 5%, 10%, and 20%) on macrophages for 24 h, the results showed that excessive mechanical loading of 20% cyclic tensile strain promoted macrophages M1 polarization (Supplementary fig. 1A), and the M1/M2 macrophages ratio increased with prolonged loading time (6 h, 12 h, and 24 h; Fig. 1B and Supplementary fig. 1B). In addition, the supernatant of macrophages with 20% cyclic tensile strain loading for 24 h promoted chondrocytes catabolism and senescence (Fig. 1C and Supplementary fig. 1C).

To investigate whether M1 macrophage-derived ectosomes induced by mechanical overloading served as important mediators leading to cartilage degradation, ectosomes derived from RAW264.7 cells stimulated by overloading (0.5 Hz, 20% cyclic tensile strain) for 24 h were collected by gradient differential centrifugation and then characterized using various techniques, including transmission electron microscopy, western blotting (WB), and ZetaView nanoparticle tracking analysis. The results showed that these spherical vesicles expressed ectosomes markers, such as Tsg101, HSP70, and CD80 (Fig. 1D, E). The mean diameter of unstimulated macrophage ectosomes (CON-Ectos) was 147.9 nm, while that of mechanically overloaded macrophage ectosomes (Tensile-Ectos) was 153.4 nm (Fig. 1F). Subsequently, Tensile-Ectos and CON-Ectos were co-cultured separately with chondrocytes. After co-culturing with Tensile-Ectos, COL2 in chondrocytes was down-regulated, whereas MMP13, P21, and P16 were up-regulated (Fig. 1G, H). These results were further confirmed in chondrocytes treated with ectosomes from mechanically overloaded primary macrophages (BMDMs) (Supplementary fig. 1D–F). In addition, by intra-articular injection of these ectosomes in DMM-OA mice (Supplementary fig. 1G), we found that Tensile-Ectos caused damage to articular cartilage in vivo, manifested by an increase in the OARSI scores and cartilage catabolism (Fig. 1I and Supplementary fig. 1H). Taken together, these findings indicated that ectosomes derived from mechanically overloaded-macrophages promoted chondrocyte degradation in vivo and in vitro, revealing the important role of macrophage ectosomes in OA progression.

### Mechanically overloaded macrophages deliver miR-350-3p to chondrocytes via ectosomes

**Fig. 2:**
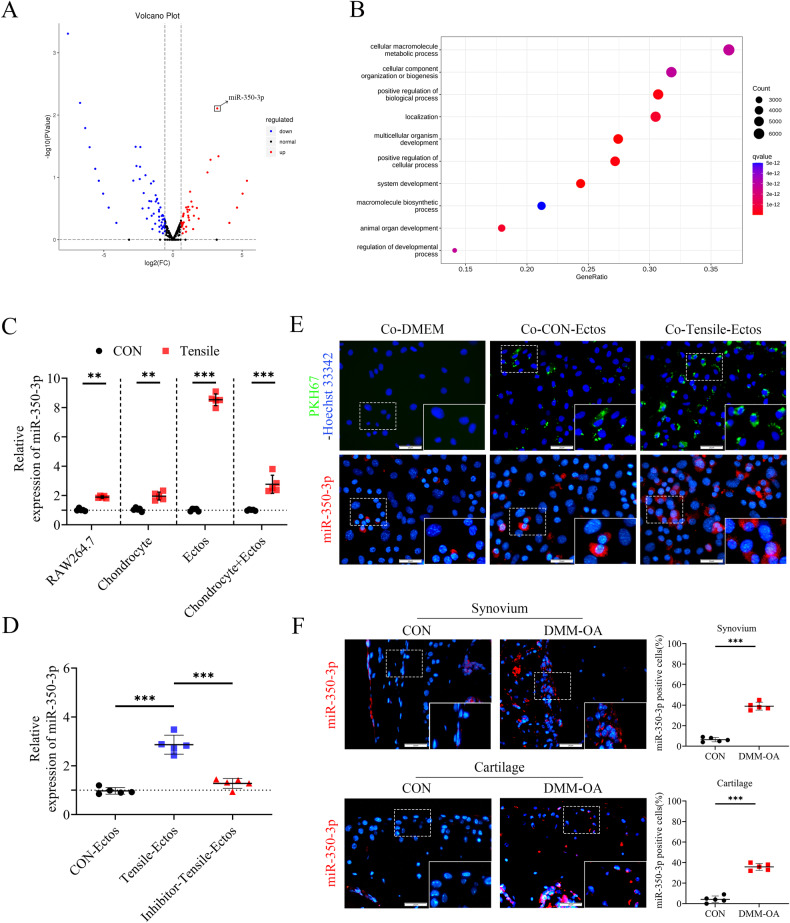
Mechanically overloaded macrophages deliver miR-350-3p to chondrocytes via ectosomes. **A** Volcano plot of upregulated and downregulated miRNAs in Tensile-Ectos compared with CON-Ectos. **B** Gene Ontology enrichment analysis of target genes predicted by differentially expressed miRNAs. **C** Quantitative PCR analysis of miR-350-3p in Raw 264.7 cells and their ectosomes, chondrocytes treated with 0.5 Hz, 20% elongation strain loading for 24 h and chondrocytes treated with CON-Ectos or Tensile-Ectos. **D** Quantitative PCR analysis of miR-350-3p in chondrocytes treated with CON-Ectos, Tensile-Ectos, Tensile-Ectos (miR-350-3p-inhibitor). **E** Fluorescent staining of chondrocytes co-cultured with purified PKH67-Ectos and fluorescence in situ hybridization of cy3-probe labeled miR-350-3p in chondrocytes treated with CON-Ectos or Tensile-Ectos. Scale bar: 25 µm. **F** Fluorescence in situ hybridization and quantification of miR-350-3p in synovial tissue and cartilage of sham operation mice (CON) and DMM mice, *n* = 5 per group. Scale bar: 25 µm. Statistical analyses were conducted by unpaired *t*-test (**F**) or two-way analysis of variance followed by Sidak’s multiple comparison test (**C**, **D**). *P < 0.05, **P < 0.01, ***P < 0.001, ns not significant.

To analyze the specific molecular mechanism of ectosomes derived from mechanically overloaded macrophages in promoting chondrocyte damage, miRNA sequencing was performed on macrophage-derived ectosomes. Compared with CON-Ectos, there were 123 up-regulated miRNAs in Tensile-Ectos, of which miR-350-3p was the most up-regulated (Fig. 2A). Gene Ontology enrichment analysis of biological processes revealed that targeted genes predicted by differentially expressed miRNAs were associated with the cellular macromolecule metabolic process (Fig. 2B).

We subsequently investigated the miR-350-3p level in macrophages and their ectosomes as well as primary chondrocytes under 20% cyclic tensile strain loading. The results showed that 20% loading led to increased miR-350-3p in cells and significant enrichment in Tensile-Ectos. Moreover, elevated miR-350-3p was also detected in chondrocytes co-cultured with Tensile-Ectos (Fig. 2C and Supplementary fig. 2A–C). To determine the origin of the elevated miR-350-3p in chondrocytes co-cultured with ectosomes, miR-350-3p inhibitor was used to modify the miR-350-3p level in mechanically overloaded macrophages, of which secreted ectosomes were collected and co-cultured with chondrocytes. Compared with the chondrocytes co-cultured with Tensile-Ectos, the level of miR-350-3p exhibited a notable reduction in chondrocytes when co-cultured with Tensile-Ectos deficient in miR-350-3p. This suggests the potential transport of miR-350-3p from macrophages to chondrocytes via ectosomes. (Fig. 2D). By labeling ectosomes with PKH67 and miR-350-3p with a probe, it was confirmed that macrophage ectosomes were readily internalized by chondrocytes, which led to miR-350-3p up-regulation (Fig. 2E). Similarly, we detected increased miR-350-3p expression in the synovium and cartilage of DMM-OA mice (Fig. 2F). These results indicated that miR-350-3p may be delivered by macrophage ectosomes and thereby participate in the regulatory mechanism of mechanical overloading in the joint microenvironment.

### MiR-350-3p promotes chondrocyte catabolism and senescence

**Fig. 3:**
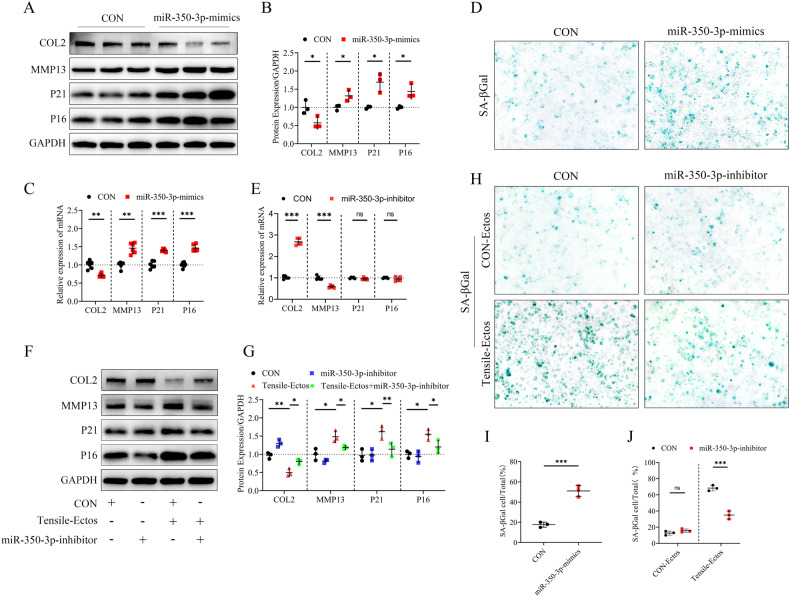
MiR-350-3p promotes chondrocyte catabolism and senescence. **A**–**C** Quantitative PCR and western blot of COL2, MMP13, P21, P16 in chondrocytes treated with miR-350-3p mimics or mimics-NC (CON). **D**, **I** Senescence-associated β-galactosidase staining of primary chondrocytes treated with miR-350p mimics, *n* = 3 per group. **E**–**G** Quantitative PCR and western blot of COL2, MMP13, P21, P16 in chondrocytes treated with inhibitor-NC (CON), miR-350-3p inhibitor, CON-Ectos and Tensile-Ectos. **H**, **J** Senescence-associated β-galactosidase staining of primary chondrocytes treated with inhibitor-NC (CON), miR-350-3p inhibitor, CON-Ectos and Tensile-Ectos, *n* = 3 per group. Statistical analyses were performed using two-way analysis of variance followed by Sidak’s multiple comparison test (**B**, **C**, **E**, **G**, **J**) or Student’s *t* test (**I**). *P < 0.05, **P < 0.01, ***P < 0.001, ns not significant.

To investigate the function of miR-350-3p in chondrocytes, we transfected primary chondrocytes with mimics NC(CON) or miR-350-3p-mimics (Supplementary Fig. 3A). QPCR and WB analysis showed that miR-350-3p-mimics treatment decreased COL2 level in chondrocytes, while also increasing MMP13, P16, and P21 levels (Fig. 3A–C). Furthermore, the enhanced expression of miR-350-3p within chondrocytes augmented the quantity of cells labeled with SA-β-galactosidase (SA-β gal), indicating the occurrence of cellular senescence.

Subsequently, we used miR-350-3p inhibitor to transfect chondrocytes co-cultured with Tensile-Ectos (Supplementary fig. 3B). The results of qPCR, WB, and SA-βGal showed that inhibiting miR-350-3p alleviated chondrocyte catabolism and senescence (Fig. 3E–H, J). In summary, inhibition of miR-350-3p partially reduced the effects of Tensile-Ectos on cartilage catabolism and senescence.

### Intra-articular injection of antagomir-350-3p partially alleviates OA progression

**Fig. 4:**
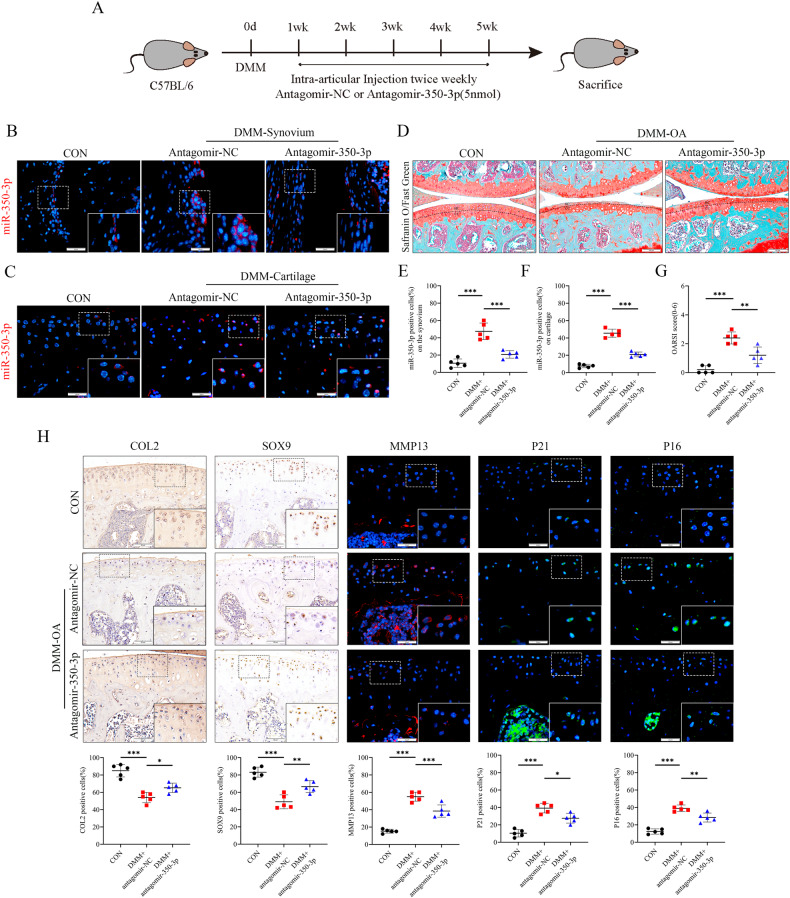
Intra-articular injection of antagomir-350-3p partially alleviates OA progression. **A** Scheme of intra-articular injection of antagomir-NC and antagomir-350-3p in DMM-OA mice. **B**, **C**, **E**, **F** Fluorescence in situ hybridization and quantification of miR-350-3p in synovium and cartilage of sham-operated mice (CON), DMM +antagomir-NC mice and DMM+antagomir-350-3p mice, *n* = 5 per group. Scale bar: 25 µm. **D**, **G** Safranin O and Fast Green staining and OARSI grades of the mice. Scale bar: 100 µm. **H** IHC/IF staining and quantification of COL2, SOX9, MMP13, P21 and P16 in cartilage from CON, DMM-OA mice treated with antagomir-NC or antagomir-350-3p. Scale bar: 50 µm. Statistical analyses were conducted using one-way analysis of variance followed by Tukey’s multiple comparison test (**E**–**G**). *P < 0.05, **P < 0.01, ***P < 0.001, ns not significant.

To assess the effectiveness of inhibiting miR-350-3p for OA in vivo, intra-articular antagomir-NC or antagomir-350-3p injections were performed one week after DMM operation in mice (Fig. 4A). Fluorescence in situ hybridization analysis revealed that the administration of antagonir-350-3p via intra-articular injection effectively blocked the increase of miR-350-3p in synovial macrophages and chondrocytes (Fig. 4B, C, E, F). Moreover, this intervention was found to mitigate the depletion of proteoglycans and the cartilage degeneration in DMM-OA mice (Fig. 4D, G). In addition, inhibiting miR-350-3p increased the COL2 and SOX9 expression in cartilage, whereas MMP13, P16, and P21 expression were decreased (Fig. 4H). These data indicated that inhibiting the miR-350-3p in joints alleviated cartilage degeneration and aging, reflecting the important role of miR-350-3p in joint stress homeostasis imbalance.

### MiR-350-3p accelerates OA development by targeting chondrocyte H3K36 methyltransferase NSD1

**Fig. 5:**
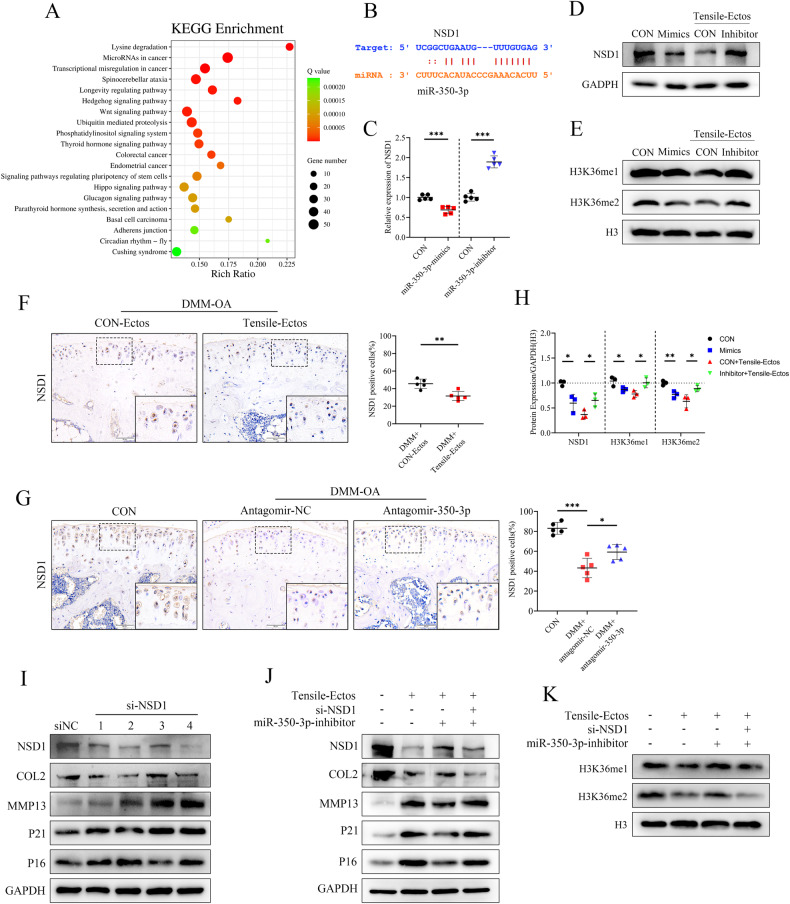
MiR-350-3p accelerates OA development by targeting chondrocyte H3K36 methyltransferase NSD1. **A** KEGG pathways enrichment analysis of the differentially genes in chondrocytes overexpressed by miR-350-3p mimics. **B** The binding site between miR-350-3p and NSD1 predicted by ENCORI database. **C**–**E**, **H** Quantitative PCR and western blot of NSD1, H3K36me1, H3K36me2 in chondrocytes treated with miR-350-3p mimics or mimics-NC (CON) and miR-350-3p inhibitor or inhibitor-NC (CON). **F**, **G** IHC staining and quantification of NSD1 in cartilage. **I** Immunoblotting of NSD1, COL2, MMP13, P16 and P21 in chondrocytes treated with si-NSD1 or si-NC. **J**, **K** Immunoblotting of NSD1, COL2, MMP13, P16, P21, H3K36me1 and H3K36me2 in chondrocytes treated with Tensile-Ectos, si-NSD1 and miR-350-3p inhibitor. Statistical analyses were conducted using Student’s *t* test (**F**), two-way analysis of variance followed by Sidak’s multiple comparison test (**C**, **H**), one-way analysis of variance followed by Tukey’s multiple comparison test (**G**). *P < 0.05, **P < 0.01, ***P < 0.001, ns not significant.

RNA sequencing on chondrocytes transfected with CON or miR-350-3p mimics was performed to analyze the specific mechanism of the effect of miR-350-3p. KEGG pathway analysis of the differentially expressed mRNAs revealed that miR-350-3p was closely associated with lysine degradation pathway (Fig. 5A). We noticed that NSD1, the most significantly down-regulated gene in this pathway, had binding sites for miR-350-3p [28], suggesting that NSD1 acted as a target of miR-350-3p (Fig. 5B). NSD1, an enzyme containing the SET domain, is responsible for methylating histones and specifically targeting the H3K36 residue. WB and qPCR analyses indicated that miR-350-3p mimics inhibited NSD1 expression in chondrocytes, resulting in a decrease in H3K36 methylation levels. In contrast, NSD1 expression and H3K36 methylation levels could be promoted by miR-350-3p inhibitor (Fig. 5C–E, H). In vivo, cartilage NSD1 was down-regulated by injecting Tensile-Ectos, but was increased by inhibiting miR-350-3p (Fig. 5F, G).

Subsequently, we investigated the role of NSD1 during OA by using siRNAs to silence chondrocyte NSD1 expression (Supplementary fig. 4A). An increase of MMP13, P16, and P21 was detected in siNSD1-treated chondrocytes, accompanied by a decrease of COL2 (Fig. 5I and Supplementary fig. 4B). In addition, NSD1 siRNA counteracted the rescue effect of miR-350-3p inhibitor on Tensile-Ectos-treated chondrocytes and accompanied by a reduction in H3K36 methylation levels (Fig. 5J, K and Supplementary fig. 4C).

Together, the increase of miR-350-3p led to the downregulation of NSD1 expression and caused decreased H3K36me1/2 levels, ultimately accelerating the progression of OA associated with mechanical loading imbalance.

## Discussion

**Fig. 6:**
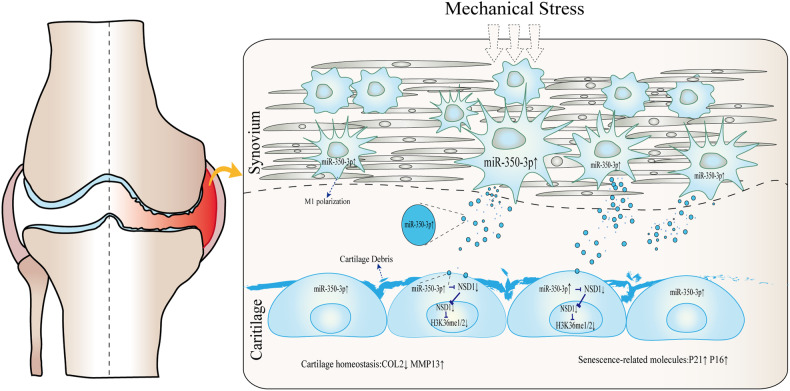
Model of macrophages accumulate M1 polarization and secrete ectosomal miR-350-3p to disrupt chondrocytes homeostasis under mechanical overloading. MiR-350-3p increases in macrophages induced by excessive mechanical loading and is delivered to chondrocytes via ectosomes. Elevated miR-350-3p in chondrocytes promotes the catabolism and aging of chondrocytes by inhibiting the expression of NSD1 and down-regulating the methylation level of H3K36, and accelerates the progression of OA.

This study elucidated the effects of mechanical overloading on macrophages and the interaction between overloaded macrophages and chondrocytes. Excessive mechanical loading induced macrophage M1 polarization, and the ectosomes secreted by polarized macrophages contained a high abundance of miR-350-3p, which mediated cartilage catabolism and aging (Fig. 6). Further investigation found that miR-350-3p was transferred from overloaded macrophages to chondrocytes via ectosomes as a carrier, resulting in a decrease in the levels of H3K36 methyltransferase NSD1 and H3K36 mono- or di-methylation in chondrocytes, promoting OA progression.

Crosstalk between synovial macrophages and chondrocytes is an important pathological mechanism of OA [29–31]. Our previous studies demonstrated that the increase of M1-polarized macrophages in OA synovium accelerates cartilage degeneration and bone formation, and the increase of pentraxin 3 in joints indirectly affected chondrocyte metabolism by regulating macrophage polarization [32, 33]. These results indicated that M1-polarized macrophages in synovitis had a profound impact on OA progression. In investigating the mechanism of interaction between macrophages and chondrocytes, some studies have shown that macrophages activated pro-inflammatory M1 polarization through metabolic reprogramming during OA, while polarized macrophages further promoted synovitis and cartilage extracellular matrix degradation by secreting a large number of proinflammatory factors and EVs, but the key initiating factors of synovial macrophage polarization and the mechanism of macrophages regulating chondrocyte metabolism remain unclear [34, 35]. In the present study, we found that mechanical overloading promoted increased M1 polarization in synovial macrophages, indicating that macrophages responded to mechanical stimulation in the environment. Furthermore, supernatants of overloaded macrophages accelerated primary chondrocytes toward catabolism and senescence, suggesting that mechanical overloading indirectly promoted chondrocyte homeostasis disruption by regulating macrophage polarization. Because mechanical stimulation is a traumatic event affecting the entire joint, stress-induced macrophage dysfunction may be an important pathological process in OA occurrence and development.

Ectosomes are derived from eukaryotic cells after direct injury, apoptosis, or activation, and have proinflammatory activity and immunomodulatory functions. Ectosomes derived from diseased joints held the potential to induce fibroblast-like synoviocytes to secrete various chemokines and cytokines [36]. In addition, ectosomes are associated with matrix metalloproteinase (MMP) expression. Ectosomes originating from monocytes and T cells specifically stimulated fibroblast-like synoviocytes to express MMP1, MMP3, MMP9, and MMP13, which was associated with the activation of inflammatory signals in the synovium [37]. However, the molecular properties and functions of ectosomes in OA are not fully understood. In our study, ectosomes derived from M1-polarized macrophages stimulated by mechanical loading induced extracellular matrix degradation and MMP expression in cartilage. In addition, chondrocytes readily absorbed PKH67-labeled ectosomes, indicating good compatibility between ectosomes and chondrocytes. These findings suggest the great potential of ectosomes in the study of degenerative diseases.

Indeed, there are multiple mechanisms for the interaction between ectosomes and target cells. Ectosomes may mediate target cell modification by presenting membrane-associated bioactive molecules that activate receptors on the target cell membrane [38]. In addition, ectosomes directly integrate the vesicle contents into the recipient cells for cell signal transduction by fusing with the receptor cell membrane, which leads to cell activation, phenotypic modification, and cell function reprograming [38]. Although the components within the donor cells are complex, the assembly of vesicle contents appears to be a selective process [39]. Studies have shown that miRNAs selectively delivered by ectosomes mediate the pathological processes of a variety of diseases, and disease progression can be effectively delayed through drug delivery systems using ectosomes as carriers [40]. MiR-350-3p was found to be present in intracellular and extracellular vesicles in plasma, and may be involved in the regulation of atherosclerosis, indicating that miR-350-3p can be transferred into EVs and participate in some pathological processes [41]. Through miRNA sequencing and analysis of ectosomes from macrophages stimulated by mechanical loading, we found that miR-350-3p was significantly increased in stimulated ectosomes, and increased miR-350-3p expression was also detected in chondrocytes co-cultured with these ectosomes. Furthermore, by co-cultivating chondrocytes with ectosomes obtained from parent cells, wherein the inhibition of miR-350-3p was implemented, we observed a substantial decrease in miR-350-3p levels within the co-cultivated chondrocytes, indicating that excessive mechanical loading promoted the transfer of miR-350-3p from macrophages to chondrocytes through ectosomes. Previous studies also reported that the intercellular metastasis of miRNAs with extracellular vesicles affected OA progression, thus it is essential to study the impact of transferred miRNA on target cells [18].

Our study showed that miR-350-3p regulates NSD1 expression in chondrocytes. NSD1 is considered to be a developmental regulatory protein that is crucial for growth and development in mammals [42]. In addition to affecting tissue growth and development, NSD1-mediated histone methylation is also associated with tumor formation [43–45]. A recent study demonstrated that NSD1 regulated chondrocyte differentiation, and its deletion led to impaired in chondrogenesis by inhibiting H3K36 methylation, suggesting the potential role of H3K36 methyltransferase NSD1 in cartilage growth, differentiation, and injury repair [26]. We found that NSD1 downregulation and reduced mono- and dimethylation levels of H3K36 were also observed in chondrocytes co-cultured with ectosomes, suggesting that synovial macrophages regulated epigenetic changes in chondrocytes through ectosomes, which may at least be partially due to the delivery of miR-350-3p.

To conclude, our study found that ectosomal miR-350-3p mediated the interaction between mechanically overloaded macrophages and chondrocytes, revealing a potential mechanism in OA development. However, at present, there are without unified standards or methods for ectosome isolation, and the molecular characteristics and functions of ectosomes in OA joints remain unclear. Therefore, future studies are worthy of exploring the potential role and mechanism of ectosomes in human OA.

## Materials and methods

### OA model and Intra-articular injection

All C57BL/6 J mice (10-week-old, male) in this study were from the Laboratory Animal Center of Southern Medical University. They were allowed to reach 12 weeks of age prior to conducting the destabilization of the medial meniscus (DMM) surgery. All animals were kept in a constant pathogen-free environment and were given a standard diet and circadian rhythm. The DMM-OA model was established under well-established anesthesia. DMM surgery, which involves the loosening of the medial meniscus, was conducted on the right knee joint of C57BL/6 J mice. The sham surgery group only incises and sutures the joint cavity. The operation is performed by a single person and remains blind all the time. In the animal part in Fig. 1A, mice were divided into two groups based on the randomization principle (*n* = 5): (1) Sham group (CON); (2) DMM-OA group. In the animal part in Fig. 1I, DMM-OA mice were divided into two groups based on the randomization principle (*n* = 5): (1) CON-Ectos group; (2) Tensile-Ectos group. One week after surgery, mice were administered intra-articular injections of either 5ul Con-Ectos (Ectosomes of unstimulated macrophage) or 5ul Tensile-Ectos (Ectosomes of macrophages stretched by 20% cyclic tensile strain) for 4 weeks (protein concentration of 200 ug/mL, twice a week). In the animal part in Fig. 4, mice were randomly divided into groups and received intra-articular injections one week after surgery(*n* = 5): (1) Sham group (CON); (2) DMM+ antagomir-NC group; (3) DMM+ antagomir-350-3p group. In the DMM+ antagomir-NC group, the mice were subjected to antagomir-NC (5 nmol) for 4 weeks (twice a week). Mice subjected to sham surgery were used in the sham group. In the DMM+ antagomir-350-3p group, the mice were treated with antagomir-350-3p (5 nmol) for 4 weeks (twice a week). After completing all intra-articular injection courses, the knee joint specimens were collected and fixed for 24 h, decalcified for 15 days and then sectioned and stained.

### Isolation and culture of bone marrow-derived macrophages

The femur and tibia were isolated from C57BL/6J mice aged 4–10 weeks. A sterile syringe was used to draw MEM-alpha medium to flush the bone marrow cavity. The cell suspension was then filtered using a sterile filter and centrifuged to remove the supernatant. Red blood cell lysis buffer was added to the cell pellet and incubated at 4°C for 10 min. Subsequently, the lysis buffer was removed and the cells were collected. BDMDs cells were cultured in DMEM with 4.5 g/L glucose, 10% fetal bovine serum and M-CSF.

### Cells cultures and co-culture

Primary chondrocytes were extracted and isolated from the articular cartilage of newborn C57BL/6J mice. The isolated and purified chondrocytes were then cultured in DMEM/F12 basic medium and added with 10% fetal bovine serum to support cell growth. Mouse RAW264.7 cells were cultured in DMEM with 4.5 g/L glucose and supplemented with 10% fetal bovine serum (Fetal bovine serum was subjected to ultracentrifugation to remove endogenous EVs). The culture conditions were 5% CO_2_ and 37 °C in a cell culture incubator. RAW264.7 cells and BMDMs were exposed to a 20% cyclic tensile strain load for a period of 24 h. After this, the supernatants were collected. These supernatants were then combined with primary chondrocytes in 12-well plates, with a mixture of supernatant and complete culture medium 1:1. This co-culture was carried out for either 24 or 48 h. In the co-culture of ectosomes with primary chondrocytes, the protein concentration of macrophage ectosomes was 20 μg/ml to stimulate primary chondrocytes for 24 h or 48 h.

### Cyclic tensile strain loading of macrophages and isolation of ectosomes

The primary chondrocytes, RAW264.7 cells and BMDMs were respectively planted on the silicon stretching chamber of the stretching plate, and each stretching hole cell was controlled at 70% density and ensured that it was adhered to a collagen-coated stretching film and then cyclic stretching load was carried out with a FLEXCEL-5000 mechanical stretching system. The condition of cyclic tensile load was 0.5 Hz and 20% cyclic stretching and time gradient stretching was carried out according to the experimental conditions. Control cells were seeded in the same stretching chamber and were not stretched. After the completion of cyclic tensile strain loading, ectosomes were collected by a standard differential centrifugation. To summarize, the cell culture medium underwent a sequential centrifugation process at 300×*g* for 10 min (at 4 °C, to eliminate the cellular components from the medium), 3000×*g* for 20 min (at 4 °C, to eliminate apoptosis bodies and cell debris). Subsequently, the supernatant underwent filtration using a 0.8-μm pore filter and centrifugation at 12,500×*g* for 90 min to gather ectosomes. After PBS washing, the ectosomes was re-suspended in PBS.

### Total RNA extraction and RT-qPCR

Primary mouse chondrocytes, macrophages, and ectosomes of macrophages were treated with TRIZOL reagent (Takara Bio Inc., Shiga, Japan) to extract total RNA. For mRNA quantification, Absorbance at 260 nm and 280 nm was used to assess RNA quantity. In order to determine the level of target mRNA, the total RNA was reverse transcribed and amplified with specific target gene primers and determined by a light cycler (Roche). To quantify miRNA levels, the miRNA was reverse transcribed using stem-loop primers and amplified and detected using specific primers for miR-350-3p.

Primer sequences were listed below:

Mouse GAPDH Forward 5’-AGGTCGGTGTGAACGGATTTG-3’

Reverse 5’-TGTAGACCATGTAGTTGAGGTCA-3’

U6 Forward 5’-CTCGCTTCGGCAGCACA-3’

Reverse 5’-AACGCTTCACGAATTTGCGT-3’

Mouse ARG Forward 5’-TTGGGTGGATGCTCACACTG-3’

Reverse 5’-GTACACGATGTCTTTGGCAGA-3’

Mouse COL2 Forward 5’-CACCCTCAAATCCCTCAACAATCA G-3’

Reverse 5’-TGTCTTTCGTCTTGCTGGTCCACC-3’

Mouse iNOS Forward 5’-GTTCTCAGCCCAACAATACAAGA-3’

Reverse 5’-GTGGACGGGTCGATGTCAC-3’

Mouse MMP13 Forward 5’-CTTCTTGTTGAGCTGGACTC-3’

Reverse 5’-CTGTGGAGGTCACTGTAGACT-3’

Mouse P21 Forward 5’-CCTGGTGATGTCCGACCTG-3’

Reverse 5’-CCATGAGCGCATCGCAATC-3’

Mouse P16^ink4a^ Forward 5’-CGCAGGTTCTTGGTCACTGT-3’

Reverse 5’-TGTTCACGAAAGCCAGAGCG-3’

mmu-miR-350-3pStem-loop primer 5’-TCGTATCCAGTGCAGGGTCCGAG GTATTCGCACTGGATACGACGAAAGT-3’

Forward primer 5’-CGCGTTCACAAAGCCCATAC-3’

### Western blot analysis

The protein in cells or ectosomes was extracted by RIPA lysate (Fdbio Science, Guangzhou, China) and ultrasonic lysis of cells and ectosomes, and protein degradation was inhibited by protein phosphatase inhibitor. The total protein levels in these samples were measured by BCA assay kit (Fdbio Science). After separation of samples by SDS-PAGE, they were transferred to a 0.22um PVDF membrane in the wet transfer method. At 4 °C on a shaker, blocking was performed on each membrane using 5% whole milk. After blocking, the membranes were further processed for immunoblotting using specific primary antibodies. Subsequently, the protein levels were measured by Fdbio-Dura ECL (Fdbio-Science) after incubation with specific secondary antibodies in the membrane for 1 h. The antibodies used in this study for western blot include: HSP70 (1:1000, Abcam, ab181606), CD81 (1:1000, Ptoteintech, 66866-1), Calnexin (1:500, Ptoteintech, 10427-2), COL2 (1:1000, Abclonal, A1560), Tsg101 (1:1000, Abcam, ab125011), MMP13 (1:1000, Abclonal, A11148), p16^INK4a^ (1:2000, Abcam, ab211542), NSD1 (1:500, Abclonal, A9981), Mono Methyl-Histone H3-K36 (1:2000, Abclonal, A22863), DiMethyl-Histone H3-K36 (1:10000, Abclonal, A22087), Histone H3 (1:2000, Abclonal, A17562), HRP-labeled goat IgG H&L (Jackson ImmunoResearch), GAPDH (1:8000, Proteintech, 60004-1-Ig), and P21 (1:1000, Abcam, ab109199).

### siRNA transfection

To inhibit NSD1 mRNA in primary chondrocytes, small interfering RNA (siRNA) was utilized. The experiment involved seeding the chondrocytes in a 6-well plate and waiting until the cell density reached 70%-90%. At that point, either 75 nM siRNA-NSD1 or siRNA negative control (Tsingke, Nanjing, China) was transfected into the cells using lipofectamine 3000. After 8 h of transfection, fresh culture medium was used and further incubated for 48 h. The following is the sequence of siRNA-NSD1 used in the research: siNSD1-1 (forward 5′-GCGGUUAAUGACUGC UCAA-3′, reverse 5′-UUGAGCAGUCAUUAACCGC-3′), siNSD1-2 (forward 5′-CU AGAUAUCCUGUCACAAA-3′, reverse 5′-UUUGUGACAGGAUAUCUAG-3′), siNSD1-3 (forward 5′-GGUUUGUCUGAGAGUGCUU-3′, reverse 5′-AAG CACUC UCAGACAAACC-3′), siNSD1-4 (forward 5′-CAGCUAUUCGGUCAGAG AA- 3′, reverse 5′-UUCUCUGACCGAAUAGCUG-3′).

### MiR-350-3p mimics and inhibitor transfection

In this study, the transfection of 50 nM miR-350-3p mimics or mimics NC (CON) using lipofectamine 3000 was performed. Subsequently, the transfected cells were incubated in fresh culture medium for an additional 48 h. Additionally, 80 nM miR-350-3p inhibitor or inhibitor control was transfected into RAW 264.7 cells and then stretched at cyclic tensile strain (0.5 Hz, 20% elongation). Primary chondrocytes were transfected using the same method and then treated with CON-Ectos or Tensile-Ectos 20ug/ml. The following is the sequence of miR-350-3p-mimics and inhibitor used in the research: mmu-miR-350-3p-mimics (forward 5′-UUCACAAAG CCCAUACACUUUC-3′, reverse 5′-AAGUGUAUGGGCUUUGUGAAUU-3′), mmu-miR-350-3p-inhibitor (5′-GAAAGUGUAUGGGCUUUGUGAA-3′), Inhibitor NC (5′-UCUACUCUUUCUAGGAGGUUGUGA-3′), and mimics-NC (forward 5′-UCA CAACCUCCUAGAAAGAGUAGA-3′, reverse 5′-UCUACUCUUUCUAGGAGG UUGUGA-3′).

### β-galactosidase (SA-β-Gal) staining and analysis

The SA-β-Gal staining kit (Beyotime) was utilized in this study to assess the activity of aging-related β-galactosidase. We followed the manufacturer’s protocols to perform cytochemical staining for SA-β-Gal. Subsequently, we randomly selected and counted the positive cells in four different regions for each treatment.

### Histological analysis

After a series of fixation, decalcification and dehydration, the tissue samples were sliced with a thickness of 4 µm. Safranin O/Fast Green stain sections were scored by two observers who were unaware of the samples’ identities. The extent of cartilage degeneration was assessed by employing the Osteoarthritis Research Society International (OARSI) scoring system for grading purposes [46].

### Immunohistochemical and immunofluorescence staining

The tissue sections underwent a process of deparaffinization and rehydration, followed by soaking in Tris-EDTA (pH 9.0) solution at a temperature of 65°C to extract antigens. Intracellular peroxidase was inactivated with 3% hydrogen peroxide and incubated with goat serum for 1 h to prepare for immunohistochemical (IHC). After washing with PBS, the sections were combined with primary antibodies for 12 h at 4°C. The IHC secondary antibody was utilized for a duration of 1 h, following which the sections underwent staining with hematoxylin and 3,3-diaminobenzidine (DAB). The immunofluorescence (IF)-conjugated secondary antibodies employing Alexa Fluor 594 and Alexa Fluor 488 (Life Technologies) were included and incubated for a duration of 1 h. Then DAPI was added dropwise and the slide was mounted. Scoring for IHC/IF was assessed by two blinded observers at three sites per joint. Antibodies used for IHC/IF staining were: P21 (1:200, Abcam, ab109199), COL2 (1:200, Abclonal, A1560), MMP13 (1:800, Abclonal, A11148), F4/80 (1:100, Santa Cruz, sc-377009), SOX9 (1:500, Proteintech, 67439-1-Ig), iNOS (1:100, Santa Cruz, sc-7271), P16^INK4a^ (1:500, Proteintech, 10883-1-AP), NSD1 (1:100, Abclonal, A9981), CD206 (1:100, Proteintech, 18704-1-AP).

### RNA fluorescence in situ hybridization

RNA fluorescence in situ hybridization kits (GenePharma, Suzhou, China) were used to determine miR-350-3p in tissues and cells. In short, tissue sections or cells are fixed and incubated overnight with CY3-labeled miR-350-3p probe at 37 °C by hybridization principle. The nuclei were labeled with DAPI.

### Ectosomes endocytosis

Purified ectosomes were labeled with the green fluorescent probe PKH-67 (Share-bio, China). The PKH67 probe was diluted to an appropriate multiple and incubated with ectosomes for 30 minutes, then co-cultured with primary chondrocytes for 6 h and stained the nuclei with Hoechst 33342. The internalization of ectosomes in chondrocytes can be observed under fluorescence microscope.

### MiRNA library construction and sequencing

The sequencing library was created using the QIAseq miRNA Library Kit (Qiagen, Frederick, MD) in adherence to the manufacturer’s instructions. Each sample was assigned an index code to distinguish its attributes. During cDNA synthesis and PCR amplification, reverse transcription primers incorporating unique molecular index were utilized to evaluate miRNA’s quantitative expression. The quality of the library was evaluated by Agilent Bioanalyzer 2100. Sequencing of the library preparation was conducted on the Illumina NovaSeq 6000 platform, and EchoBiotech Co. Ltd., Beijing, P. R. China generated pair-end readings.

### Quantification and analysis of miRNA

Clean Reads was compared against a variety of databases, including GtRNAdb, Silva, Repbase and Rfam, using Bowtie software. Then filter out duplicate sequences and ncRNAs. In order to detect novel and known miRNAs, the reads that were left were utilized and compared to the known miRNAs in the Human Genome (GRCh38) and miRbase. The UMI count expression matrix of miRNA was standardized to TPM and subsequently determined as relative log expression by utilizing the EdgeR package. To conduct differential expression analysis between the CON-Ectos group and the Tensile-Ectos group, the Mann Whitney *U* test was performed. Gene Ontology enrichment analysis was carried out with the topGO R package to investigate the target genes of the differentially expressed miRNAs.

### Statistical analysis

Statistical analysis and graph generation were carried out using Adobe Photoshop 2020 and GraphPad Prism 8.0.2. To compare two distinct groups, we employed the independent sample t-test technique, whereas the analysis of ANOVA was utilized to assess comparisons among several groups. When P-values < 0.05, the results are considered statistically significant.

### Supplementary information

Original Data File

Supplementary Materials

## Data Availability

Supplementary figures are included in Supplementary Materials. Original Western blots are included in Original Data File.
