# Supplementary figures and images for: Macrophage-derived ectosomal miR-350-3p promotes osteoarthritis progression through downregulating chondrocyte H3K36 methyltransferase NSD1
Source: Cell Death Discov. 2024 May 8;10:223. doi: 10.1038/s41420-024-01986-5 (PMC11078928; doi:10.1038/s41420-024-01986-5)

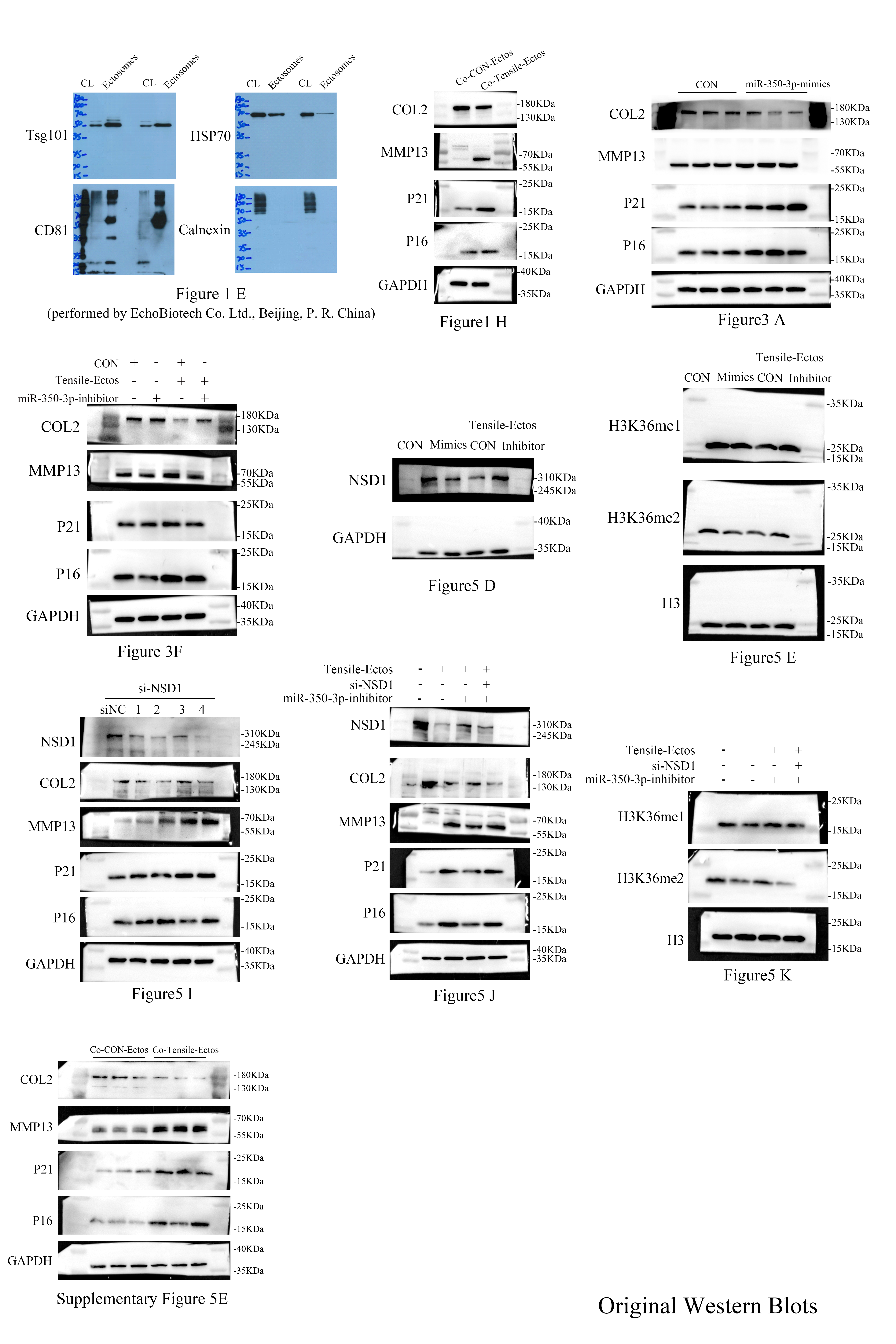

Supplement: Supplementary file 1 — Original Data File [file 41420_2024_1986_MOESM1_ESM.doc]
