# Supplementary material for: Macrophage-derived ectosomal miR-350-3p promotes osteoarthritis progression through downregulating chondrocyte H3K36 methyltransferase NSD1
Source: Cell Death Discov. 2024 May 8;10:223. doi: 10.1038/s41420-024-01986-5 (PMC11078928; doi:10.1038/s41420-024-01986-5)
Supplement: Supplementary file 2 — Supplementary Materials [file 41420_2024_1986_MOESM2_ESM.pdf]

Supplementary Materials

Macrophage-derived ectosomal miR-350-3p promotes osteoarthritis progression through downregulating chondrocyte H3K36 methyltransferase NSD1

Rengui Lin[1,2,3,4#], Jianbin Yin[1,2,3,4#], Jialuo Huang[1,2,3,4#], Liping Zou[3,4#], Liangliang Liu[1,2,3,4], Wen Tang[1,2,3,4], Hongbo Zhang[1,2,3,4], Lingfeng Yang[1,2,3,4], Yu Zhang[1,2,3,4], Guangming Li[1,2,3,4], Guiqing Wang[5], Daozhang Cai[1,2,3,4], Haiyan Zhang[1,2,3,4*], Yanli Liu[3,4*], Yan Shao[1,2,3,4*]

[1]Department of Joint Surgery, Center for Orthopaedic Surgery, The Third Affiliated Hospital of Southern Medical University, Guangzhou, China

[2]Department of Orthopedics, Orthopedic Hospital of Guangdong Province, Academy of Orthopedics · Guangdong Province, The Third Affiliated Hospital of Southern Medical University, Guangzhou, China

[3]The Third School of Clinical Medicine, Southern Medical University, Guangzhou, China

[4]Guangdong Provincial Key Laboratory of Bone and Joint Degeneration Diseases, Guangzhou, China

[5]The Sixth Affiliated Hospital of Guangzhou Medical University, Qingyuan People's Hospital, orthopedics department, Qingyuan, Guangdong, China

Supplementary Figure 1 to 4

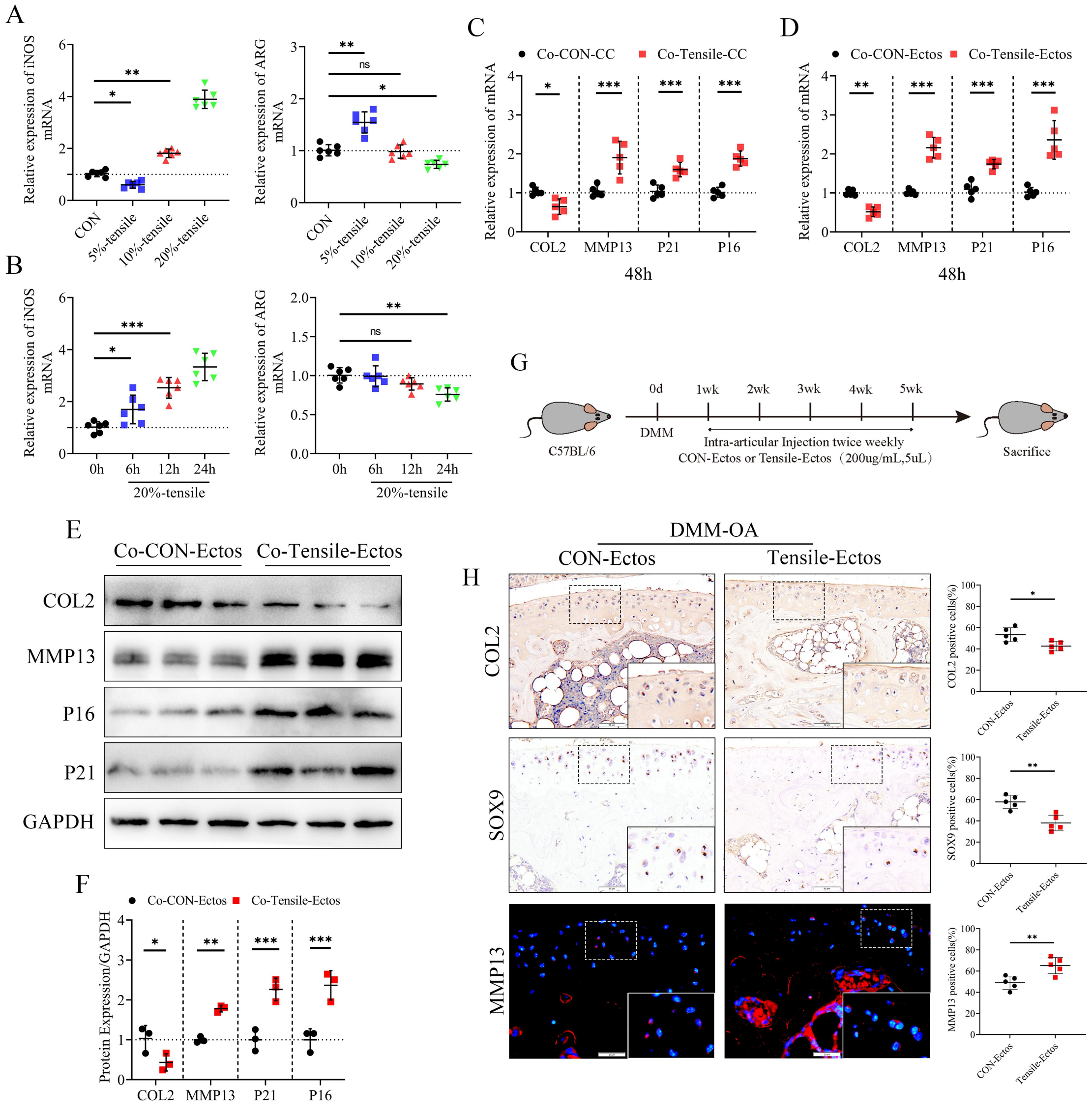

Supplementary figure 1

Quantitative PCR analysis of iNOS and ARG in BMDMs treated with 5%, 10% and 20% elongation strain loading, n=6 per point. (B) Quantitative PCR analysis of iNOS and ARG in BMDMs treated with 0.5Hz, 20% elongation strain loading for 0, 6, 12 and 24 hours, n=6 per point. (C) Quantitative PCR analysis of MMP13, COL2, P21 and P16 in chondrocytes co-cultured with supernatants of overloaded-BMDMs for 48h, n=5 per group. (D-F) Quantitative PCR and western blot of MMP13, COL2, P21 and P16 in chondrocytes treated with ectosomes from mechanically overloaded primary macrophages (BMDMs). (G) Scheme of intra-articular injection of CON-Ecots and Tensile-Ecots in DMM-OA mice. (H) IHC/IF staining and

quantification of COL2, SOX9, MMP13 in cartilage from DMM-OA mice treated with CON-Ectos or Tensile-Ectos. Statistical analyses were conducted using unpaired t-test (H), one-way analysis of variance followed by Dunnett's multiple comparison test (A, B) or two-way analysis of variance followed by Sidak's multiple comparison test (C, D, F). *P<0.05, **P<0.01, ***P<0.001, ns not significant.

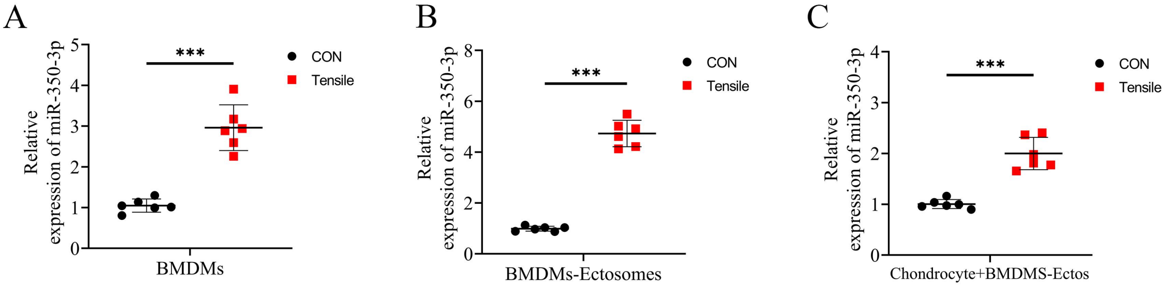

Supplementary figure 2

(A) Quantitative PCR analysis of miR-350-3p in BMDMs treated with 0.5Hz, 20% elongation strain loading for 24h. (B) Quantitative PCR analysis of miR-350-3p in ectosomes from mechanically overloaded primary macrophages (BMDMs). (C) Quantitative PCR analysis of miR-350-3p in chondrocytes treated with CON-Ectos or Tensile-Ectos from BMDMs. Statistical analyses were conducted using Student's t-test. ***P<0.001.

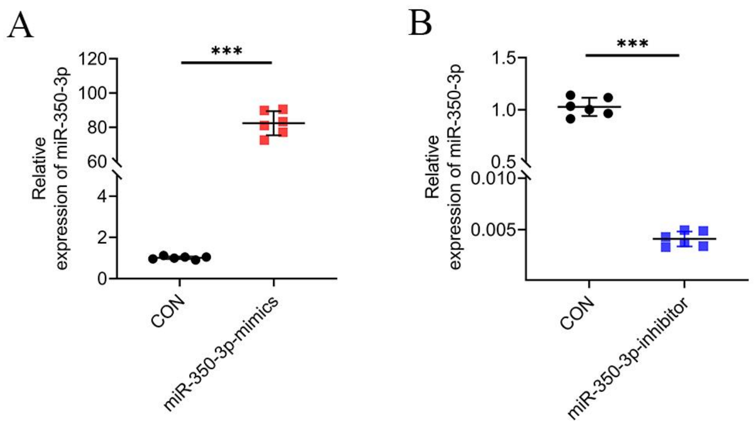

Supplementary figure 3

(A) Relative miRNA expression levels of miR-350-3p in chondrocytes treated with miR-350-3p mimics or mimics-NC (CON). (B) Relative miRNA expression levels of miR-350-3p in chondrocytes treated with miR-350-3p inhibitor or inhibitor-NC (CON). Statistical analyses were conducted using Student's t-test. ***P<0.001.

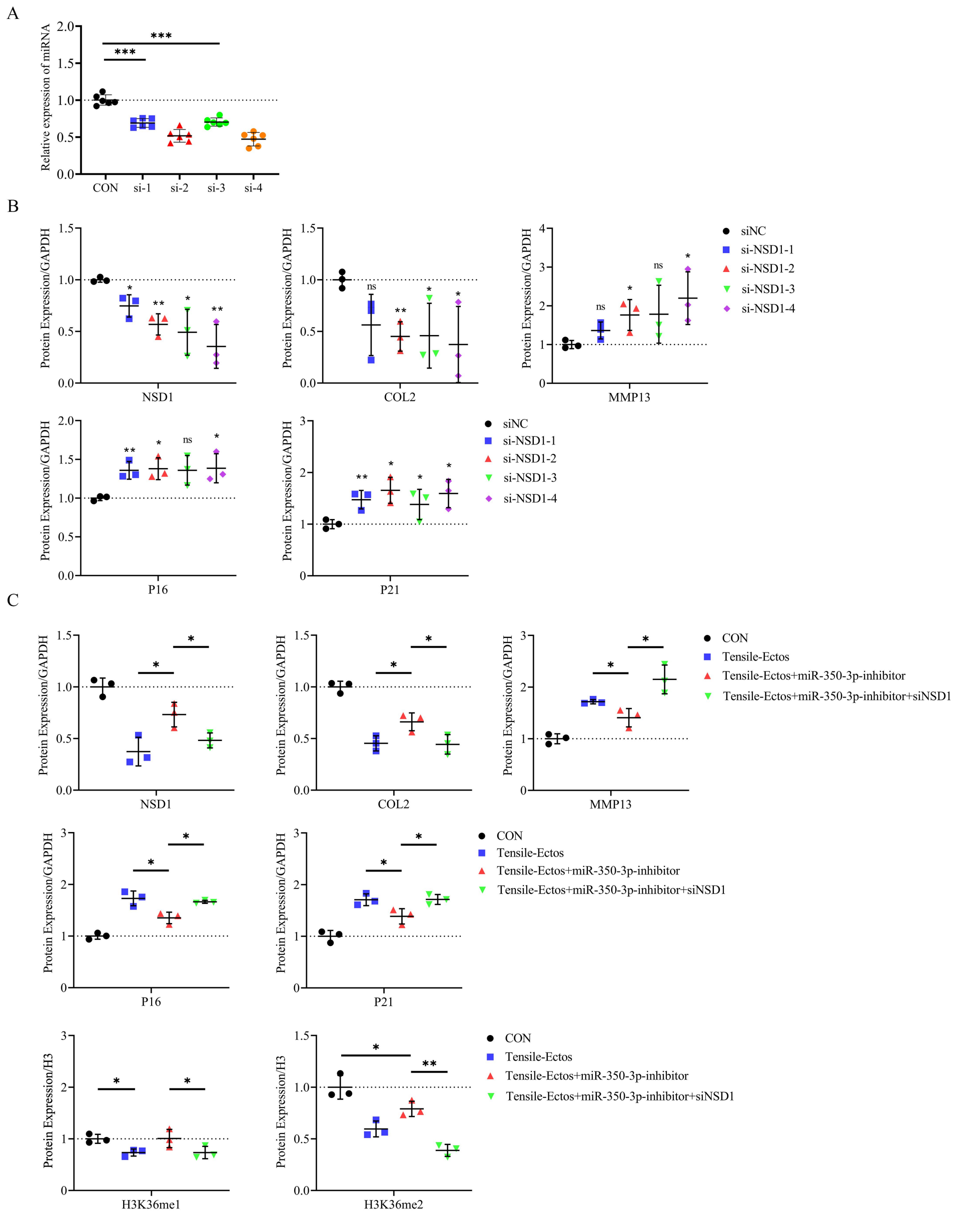

Supplementary figure 4

(A) Relative mRNA expression levels of NSD1 in chondrocytes treated with miR-350-3p mimics or mimics-NC(CON) and miR-350-3p inhibitor or inhibitor-NC(CON). (B) Relative protein quantification of NSD1, COL2, MMP13, P16 and P21 in chondrocytes treated with si-NSD1 or si-NC. (C) Relative protein quantification of NSD1, COL2, MMP13, P16, P21, H3K36me1 and H3K36me2 in chondrocytes treated with Tensile-Ectos, si-NSD1 and miR-350-3p inhibitor. Statistical analyses were conducted using one-way analysis of variance followed by Tukey's multiple comparison test (A-C). *P<0.05, **P<0.01, ***P<0.001, ns not significant.
